# Subjective psychiatric symptoms in post-acute autoimmune encephalitis: findings from the Australian autoimmune encephalitis consortium

**DOI:** 10.1007/s00415-025-13353-0

**Published:** 2025-09-29

**Authors:** Katherine Y. Ko, Christina Kazzi, Nabil Seery, Sarah Griffith, Robb Wesselingh, Tiffany Rushen, Tracie H. Tan, Hannah Ford, Catherine Meade, Marie F. O’Shea, Laurie McLaughlin, Genevieve Skinner, Mirasol Forcadela, Amy Halliday, Andrew Duncan, Ernest G. Butler, Anneke Van Der Walt, Tomas Kalincik, Wendyl D’Souza, Udaya Seneviratne, Katherine Buzzard, Richard Macdonell, Sudarshini Ramanathan, Stefan Blum, Jayashri Kulkarni, Stephen W. Reddel, Todd A. Hardy, Helmut Butzkueven, Terence J. O’Brien, Rubina Alpitsis, Charles B. Malpas, Mastura Monif

**Affiliations:** 1 Department of Neurosciences, School of Translational Medicine, Faculty of Medicine, Nursing and Health Sciences, Monash University, Level 6, Alfred Centre, 99 Commercial Road, Melbourne, VIC 3004 Australia; 2 Department of Neurology, Alfred Health, Level 6, Alfred Centre, 99 Commercial Road, Melbourne, VIC 3004 Australia; 3 Neuroimmunology Centre, Department of Neurology, Royal Melbourne Hospital, 300 Grattan Street, Parkville, VIC 3050 Australia; 4 CORe, Department of Medicine, The University of Melbourne, Parkville, VIC 3050 Australia; 5 Melbourne School of Psychological Sciences, The University of Melbourne, Parkville, VIC 3050 Australia; 6 Department of Neurosciences, St Vincent’s Hospital, 41 Victoria Parade, Fitzroy, VIC 3065 Australia; 7 Department of Neurology, Princess Alexandra Hospital, 199 Ipswich Road, Woolloongabba, QLD 4102 Australia; 8 School of Medicine, The University of Queensland, 20 Weightman Street, Herston, QLD 4006 Australia; 9 Department of Neurology, Austin Health, 145 Studley Road, Heidelberg, VIC 3084 Australia; 10 Department of Neurology, Peninsula Health, 49 Cranbourne Road, Frankston, VIC 3199 Australia; 11 Department of Neurosciences, Monash Health, 246 Clayton Road, Clayton, VIC 3168 Australia; 12 Department of Neurosciences, Eastern Health Clinical School, Monash University, 8 Arnold Street,, Box Hill, VIC 3128 Australia; 13 Department of Neurology and Concord Clinical School, Concord Repatriation General Hospital, Hospital Road, Concord, NSW 2139 Australia; 14 Translational Neuroimmunology Group, Kids Neuroscience Centre, Faculty of Medicine and Health, University of Sydney, Science Road, Camperdown, NSW 2050 Australia; 15 Brain and Mind Centre, Faculty of Medicine and Health, Sydney Medical School, University of Sydney, Science Road, Camperdown, NSW 2050 Australia

**Keywords:** Autoimmune encephalitis, Psychopathology, Neuropsychology, Neuroimmunology, Patient-reported outcomes

## Abstract

**Background and objectives:**

This study examined self-reported psychopathology in patients with autoimmune encephalitis (AE), and explored their relationship with clinical outcomes as measured by the modified Rankin Scale (mRS) and Clinical Assessment Scale in Autoimmune Encephalitis (CASE) scores.

**Methods:**

Eighty-seven AE patients (49.43% female, mean age = 54.47 years, *SD* = 17.84) from the Australian Autoimmune Encephalitis Consortium completed the SPECTRA-Indices of Psychopathology, time-matched with CASE and mRS scores within six months of the assessments.

**Results:**

On average, assessments occurred 12 months post-onset. Median CASE and mRS scores were 2, with 84.13% of patients scoring mRS ≤ 2. Mean SPECTRA scores were significantly elevated across multiple domains, indicating greater psychopathology, particularly in depression, post-traumatic stress, suicidal ideation, and cognitive functioning. Patients with seronegative AE exhibited more severe psychopathology compared to those with anti-*N*-methyl-D-aspartate receptor (NMDAR) and anti-leucine-rich glioma-inactivated 1 (LGI1) subtypes. Psychiatric diagnoses prior to AE onset were associated with worse outcomes, compared to those with no pre-existing psychiatric conditions and those diagnosed post-onset. The mRS and CASE scores each explained only a small amount of variance (7–26%) across SPECTRA domains, and adding the other score to the model provided only little improvement.

**Discussion:**

AE patients exhibit significant psychopathology across multiple domains, with significant internalising symptoms—including depression, post-traumatic stress, and suicidal ideation—as well as cognitive concerns, highlighting the considerable psychiatric burden in this population. Our findings highlight the need for systematic, timely assessments of psychological symptoms in AE, and the use of sensitive, comprehensive instruments to capture the full spectrum of their psychopathology.

**Supplementary Information:**

The online version contains supplementary material available at 10.1007/s00415-025-13353-0.

Autoimmune encephalitides (AE) are a rare, complex group of diseases characterised by immune-mediated inflammation of the brain, often triggered by antibodies targeting neuronal or synaptic proteins [1]. AE subtypes are identified according to the autoantibodies, such as anti-*N*-methyl-D-aspartate receptor (NMDAR) encephalitis, anti-leucine-rich glioma-inactivated protein 1 (LGI1) encephalitis, anti-contactin-associated protein-like 2 antibody (CASPR2), and anti-α-amino-3-hydroxy-5-methyl-4-isoxazolepropionic acid receptor encephalitis (AMPAR) encephalitis [2]. Approximately half of all AE cases are seronegative, where no known specific antibodies are detected [3, 4].

Psychiatric manifestations in AE are common and can be associated with significant morbidity [5, 6]. They may precede or coincide with neurological symptoms/signs, emerge after a prolonged period, or, in rare cases, present as isolated psychiatric syndromes [7–9]. Although limited, current evidence suggests that each AE subtype may have a distinct psychiatric phenotype [10], with psychosis, mood dysfunction, and acute behavioural and personality changes frequently observed across subtypes [7, 11]. NMDAR AE, the most common subtype, often presents with prominent psychiatric symptoms such as psychosis and behavioural disturbances, typically preceding the onset of neurological features [8, 12, 13]. In LGI1 AE, psychiatric manifestations are less widely reported, though some patients present acutely with mood disturbances and psychotic symptoms, such as hallucinations, paranoid delusions, disorganised speech, and apathy [13–15]. AMPAR AE has been associated with behavioural abnormalities resembling rapid-cycling bipolar disorder, including mood fluctuations, agitation, lethargy, and psychosis, primarily reported in case studies [16, 17]. Additionally, psychotic symptoms and mood disturbances may occur in acute cases of CASPR2 and seronegative AE, although this is infrequently reported [10, 18, 19].

Monitoring psychopathology in AE is particularly challenging due to the limitations of standard assessment tools. The modified Rankin Scale (mRS)—originally developed for evaluating stroke patient outcomes—is often used to evaluate disease severity and outcomes in AE patients [20]; however, it fails to sufficiently capture psychiatric and cognitive symptoms. The Clinical Assessment Scale in Autoimmune Encephalitis (CASE) was specifically designed for evaluating AE [21], yet its emphasis on acute symptoms limits its sensitivity to long-term cognitive and behavioural deficits [22]. Neither tool fully represents the broad spectrum of psychopathology observed in AE, highlighting the need for more appropriate assessments to guide treatment and improve outcomes.

The aim of this study is to characterise the psychopathology profile of a cohort of post-acute AE patients by assessing their self-reported symptoms and examining the relationship between these symptoms and clinical outcomes, as measured by the mRS and CASE scores.

## Methods

### Participants

Participants were consecutively recruited from outpatient neurology clinics at nine hospitals across Australia, including seven major metropolitan hospitals in Melbourne, Victoria (Alfred Health, St Vincent’s Hospital, Royal Melbourne Hospital, Monash Medical Centre, Eastern Health, Austin Health, and Peninsula Health), one in Brisbane, Queensland (Princess Alexandra Hospital), and one in Sydney, New South Wales (Concord Repatriation General Hospital). This study was conducted as part of the larger Australian Autoimmune Encephalitis Consortium Study. Eligibility criteria included (1) meeting the criteria for AE as outlined by Graus et al. [23]; (2) being at least 6 months post-diagnosis of AE; and (3) having English as the primary language. Exclusion criteria comprised a confirmed or suspected diagnosis of a neurodegenerative disorder (e.g., Alzheimer’s disease) or a history of developmental language disorder or intellectual disability. Within the seronegative cohort, diagnostic classification was based on the 2016 Graus criteria, whereby ‘possible’ AE refers to a subacute onset of cognitive, psychiatric, or neurological symptoms with at least one supportive feature (e.g., seizures, CSF pleocytosis, MRI changes); ‘probable’ AE indicates additional supportive evidence such as characteristic MRI, EEG, or clinical syndromes; and ‘definite’ AE requires the presence of a well-characterised neuronal antibody or histopathological confirmation.

Ethical approval was granted by the central Human Research Ethics Committee at Alfred Health (HREC/17/Alfred/168). Written informed consent was obtained from all participants. In cases where participants could not provide consent, approval was obtained from their responsible caregiver or legal guardian.

### Procedure

Participants completed patient-reported outcome measures (e.g., questionnaires) either during a comprehensive neuropsychological assessment in a controlled clinical setting, or independently, either by mail or in person at the clinic. A total of 87 participants completed the SPECTRA Indices of Psychopathology. Clinical outcomes data, including CASE and mRS scores, were collected at four key time points: initial admission, discharge, 12 month follow-up, and final follow-up. These scores were either retrospectively assigned by neurologists (N.S., R.W.) or prospectively recorded by clinicians during routine outpatient reviews.

### Measures

#### Psychopathology spectra

The SPECTRA Indices of Psychopathology (hereinafter referred to as SPECTRA) is a 96-item self-report measure designed to assess transdiagnostic dimensions of psychopathology within a hierarchical framework [24]. At its core is the General Psychopathology Index (GPI), which captures overall symptom burden and psychological distress. The SPECTRA includes three broad-spectrum indices: Internalising (INT), Externalising (EXT), and Reality Impairing (RIM). The Internalising spectrum reflects symptoms associated with mood and anxiety disorders, including distress, depression, and somatisation. The Externalising spectrum measures traits related to impulsivity, aggression, and behavioural dysregulation, often associated with conduct and substance use disorders. The Reality Impairment spectrum captures symptoms characteristic of psychotic-spectrum disorders, such as cognitive disorganisation, delusional ideation, and perceptual disturbances. These broad-spectrum indices are further divided into 12 lower-order clinical domains, offering greater dimensional specificity: Depression (DEP), Anxiety (ANX), Social Anxiety (SOC), and Post-Traumatic Stress (PTS)—under the Internalising spectrum; Alcohol Problems (ALC), Drug Problems (DRG), Antisocial Behaviour and Impulsivity (ANTI), and Severe Aggression (AGG)—under the Externalising spectrum; and Psychosis (PSY), Paranoid Ideation (PAR), Manic Activation (MAN), and Grandiose Ideation (GRA)—under the Reality Impairing spectrum. The SPECTRA also includes three supplemental scales: Cognitive Concerns (COG), Psychosocial Functioning (PF), and Suicidal Ideations (SUI). We selected the SPECTRA as it provides a brief, transdiagnostic assessment across multiple symptom domains, making it well-suited to the heterogeneous psychiatric presentations in AE. Its dimensional, spectra-based structure—aligned with the Hierarchical Taxonomy of Psychopathology (HiTOP) model [25]—allows for reliable characterisation of complex, overlapping symptoms, therefore reducing diagnostic noise while minimising patient burden in a cognitively fatigued population.

The SPECTRA uses a standardised five-point Likert-type scale (0 = “Not at all true” to 5 = “Completely true”) to rate symptom frequency and severity. Total and subscale scores are summed and converted to *T*-scores, with higher scores indicating greater symptomatology. SPECTRA scores were interpreted using normative data derived from a census-matched nationally representative healthy sample, which are age- and sex-adjusted in accordance with the test’s standardisation. *T*-scores are categorised into six levels: Low (≤ 45), Average (46–56), Mild (57–63), Moderate (64–69), Severe (70–89), and Extreme (≥ 90). In this study, we dichotomised symptom severity into ‘not elevated’ (< 70) and ‘elevated’ (≥ 70). The clinical scales showed strong convergent validity, with average correlations around 0.67-0.68 with similar measures, while the supplemental scales demonstrated moderate to strong associations with cognitive and personality measures, supporting their reliability and validity [26]. Internal consistency (Cronbach α) in the current sample was excellent (α = 0.90).

#### Functional outcomes

##### Modified rankin scale (mRS)

The Modified Rankin Scale (mRS) is a widely used, clinician-rated measure of functional disability and dependence in activities of daily living [20]. It rates disability on a seven-point scale from 0 (no symptoms) to 6 (death), with higher scores indicating greater functional impairment. The mRS is a well-established tool in neurological research and clinical practice, commonly used to assess outcomes following conditions, such as stroke and autoimmune encephalitis. It has demonstrated strong test–retest (weighted kappa = 0.81–0.95) and good construct validity (*r*s = 0.44–0.67), correlating well with other measures of disability and quality of life.

##### Clinical assessment scale in autoimmune encephalitis (CASE)

The Clinical Assessment Scale in Autoimmune Encephalitis (CASE) is a nine-item clinician-rated measure designed to assess the severity of AE in terms of seizures, memory dysfunction, language function, psychiatric symptoms, consciousness, dyskinesia/dystonia, gait and ataxia, limb weakness, and brainstem dysfunction [21]. Scores range from 0 to 27, with higher scores reflecting greater symptom severity. The CASE has demonstrated strong validity and reliability in AE populations, showing good inter-rater reliability (weighted kappa > 0.80) and sensitivity to clinical change.

### Data analysis

Analyses were conducted using R (version 4.5.1) and GraphPad Prism (version 10.4.0). Statistical significance was set at *p* < 0.05. Missing data were handled with pairwise deletion. All analyses were adjusted for biological sex and age. We used a Kruskal–Wallis test to assess differences across subtypes. Welch’s independent samples *t*-tests were conducted to compare psychopathology between patients with and without psychiatric comorbidities, as well as those with psychiatric diagnoses prior to AE onset versus those who were diagnosed post-onset. Spearman rank correlation coefficients were used to investigate the relationship between SPECTRA scores and the CASE score. SPECTRA scores were also analysed using on two dichotomised outcome categories of the mRS: “Good” (≤ 2) versus “Poor” (> 2) and “Good” (≤ 1) versus “Poor” (> 1). Hierarchical linear regression models were conducted to assess the contribution of mRS and CASE score in explaining SPECTRA outcomes. The clinical outcome scores were entered hierarchically: mRS was entered in the first step, and CASE was entered in the second step.

## Results

Our cohort consisted of 87 individuals with a mean age of 54.47 years (*SD* = 17.83, range = 18–84), with 49.43% identifying as female. On average, there was a 1.53-month delay between symptom onset and hospital admission (range = 0–12 months). The most common AE subtype was seronegative (33.33%), followed by LGI-1 (16.09%) and NMDAR (16.09%). Approximately 68% of patients were on at least one anti-seizure medication, and the most frequently used treatment was Intravenous Immunoglobulin (IVIg), followed by pulse steroids (66.67%).

For the demographic profile of the sample (*N*=87), refer to Table 1.

**Table 1:** Cohort demographics

Characteristics	
Age, years (*M*, *SD* [range])	54.5, 17.8 [18–84]
Biological sex, female (*N* [%])	43 [49.4]
Months between symptom onset to hospital admission (*M*, *SD* [range])	1.5, 3.1 [0–12]
ICU admissions, *y* (*N* [%])	27 [31.03]

Antibody status, *y* (*N* [%])	
Seropositive	
Anti NMDAR *ab*-mediated AE	21 [24.1]
Anti LGI-1 *ab*-mediated AE	17 [19.5]
IGLON5 AE	3 [3.5]
Associated with onconeuronal antibodies	3 [3.5]
Anti AMPAR *ab*-mediated AE	1 [1.2]
Seronegative, *y* (*N* [%])	
Seronegative limbic AE	11 [12.6]
Possible	25 [28.7]
Probable	6 [7]
Time of SPECTRA administration from disease diagnosis in months, (*M*, *SD* [range])	69.1, 49.8 [6–231]
Treatment line,* y* (*N* [%])	
First line	35 [40.2]
Second line	26 [29.9]
Maintenance Therapy	10 [11.5]
None	16 [18.4]
ASM use, *y* (*N* [%])	
0 ASM	27 [31.03]
1 ASM	18 [20.7]
2 + ASM	41 [47.1]
Immunotherapy, *y* (*N* [%])	
IVIg	61 [70.1]
Pulse steroids	58 [66.7]
Steroids	51 [58.6]
Rituximab	37 [42.5]
Plasma exchange	18 [20.7]
Cyclophosphamide	9 [10.3]
Other	29 [33.3]
None	2 [2.3]
mRS at 12 months (*N* [%])	
1	22 [25.3]
2	21 [24.1]
3	10 [11.5]
mRS at discharge (*N* [%])	
1	6 [6.9]
2	19 [21.8]
3	20 [23]
4	8 [9.2]
5	2 [2.3]
Time since onset (*N* [%])	
0–1 month	2 [2.3]
2–6 months	2 [2.3]
7–12 months	4 [4.6]
13–24 months	9 [10.4]
25–36 months	7 [80.5]
37–48 months	6 [6.9]
> 48 months	57 [65.5]
Time from onset to treatment (*N* [%])	
0–1 month	65 [74.7]
2–6 months	13 [14.9]
7–12 months	3 [3.5]
13–24 months	0 [0.0]
25–36 months	3 [3.5]
37–48 months	1 [1.2]
> 48 months	2 [2.3]
Psychiatric comorbidities pre-onset, *y* (*N* [%])	
Depression	18 [20.7]
Anxiety	13 [14.9]
ADHD	1 [1.2]
PTSD	1 [1.2]
Personality disorder	1 [1.2]
Psychiatric comorbidities post-onset, *y* (*N* [%])	8 [9.2]
Use of antidepressants on admission, *y* (*N* [%])	16 [18.4]
Venlafaxine	6 [6.9]
Escitalopram	5 [5.8]
Mirtazapine	5 [5.8]
Current use of antidepressants, *y* (*N* [%])	16 [18.4]
Venlafaxine	6 [6.9]
Escitalopram	5 [5.8]
Mirtazapine	5 [5.8]
Use of antipsychotics on admission, *y* (*N* [%])	20 [23]
Olanzapine	12 [13.8]
Quetiapine	10 [11.5]
Risperidone	8 [9.2]
Current use of antipsychotics, *y* (*N* [%])	20 [23]
Olanzapine	12 [13.8]
Quetiapine	10 [11.5]
Risperidone	8 [9.2]

*ADHD* attention-deficit/hyperactivity disorder, *ASM* antiseizure medication, *ICU* intensive care unit, *IVIg* intravenous immunoglobulin, *PTSD* post-traumatic stress disorder

### Differences between patient scores versus normative data

**Fig. 1:**
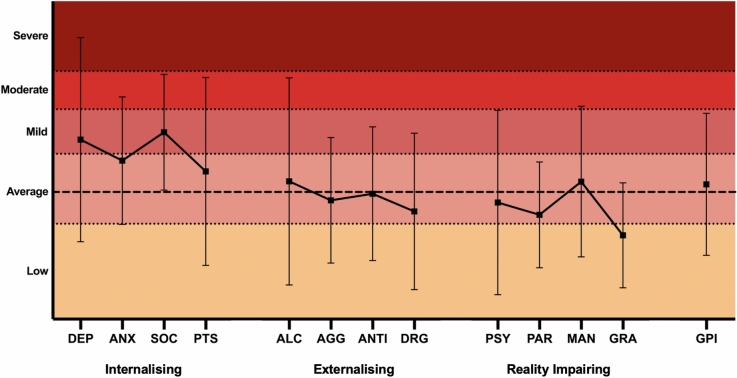
Distribution of SPECTRA Clinical Scale *T*-Scores: The dashed line at *T* = 50 represents the normative mean, and error bars show 95% confidence intervals (CIs) around the mean. Shaded areas indicate five levels: Low (≤ 45), Average (46–56), Mild (57–63), Moderate (64–69), and Severe (70–89). *AGG* severe aggression, *ALC* alcohol problems, *ANTI* antisocial behaviour, *ANX* anxiety, *DEP* depression, *DRG* drug problems, *GPI* general psychopathology index, *GRA* grandiose ideation, *MAN* manic activation, *SOC* social anxiety, *PAR* paranoid ideation, *PSY* psychosis, *PTS* posttraumatic stress

**Fig. 2:**
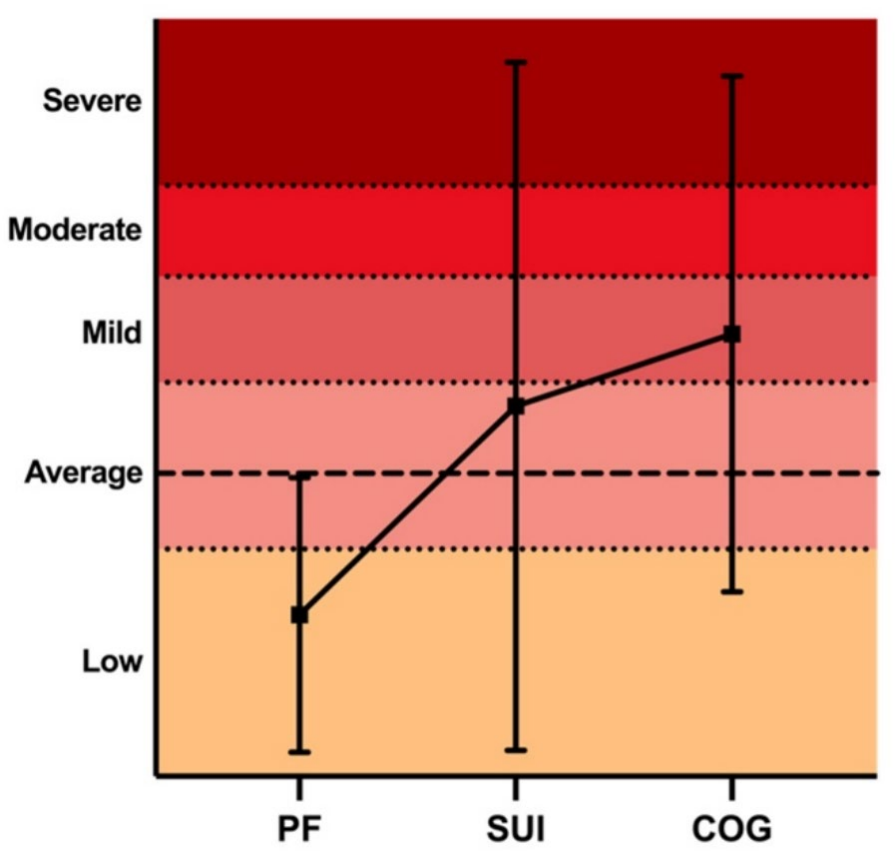
Distribution of SPECTRA Supplementary Scale *T*-Scores: The dashed line at *T* = 50 represents the normative mean, and error bars show 95% confidence intervals (CIs) around the mean. Shaded areas indicate five levels: Severe (70–89), Moderate (64–69), Mild (57–63), Average (46–56), and Low (≤ 45). *COG* cognitive concerns, *PF* psychosocial functioning, *SUI* suicidal ideation

Patients did not differ significantly from the normative mean in Global Psychopathology but showed significantly elevated symptomatology within the Internalising spectrum. In contrast, the Externalising and Reality Impairing spectra did not show significant elevation compared to the normative mean. Self-reported scores for Cognitive Concerns, Depression, Post-Traumatic Stress, and Suicidal Ideation were notably higher than normative levels. However, no significant concerns were observed for Antisocial Behaviour, Psychosocial Functioning, Grandiosity, Alcohol Problems, Paranoid Ideation, Social Anxiety, Severe Aggression, Drug Problems, Anxiety, or Psychosis. Table 2 displays the descriptive statistics and overall classification scores for patients categorised as ‘not-elevated’ and ‘elevated’ on the SPECTRA. Figures 1, 2 illustrate the distribution of SPECTRA *T*-Scores.

**Table 2:** Descriptive Statistics, Overall Classification Scores, and One-Sample *t*-test Results Comparing SPECTRA Scores to Normative Data (*T* = 50) across Spectra Indices, Clinical Scales, and Supplemental Scales

				*Classification, N [%]*			
		*M*	*SD*	‘Not Elevated’ *T* < *70*	‘Elevated’ *T* ≥ *70*	*t*	Cohen’s *d* (95% CI)
SPECTRA Indices	Internalising	55.2	11.45	79 [91]	8 [9]	4.3***	0.46 (0.23, 0.68)
	Externalising	46.6	11	85 [98]	2 [2]	−3.8***	0.40 (0.18, 0.62)
	Reality impairing	46.5	8.6	86 [99]	1 [1]	−3.2***	0.35 (0.13, 0.56)
	Global	52	12	84 [97]	3 [3]	0.7	0.08 (−0.13, 0.38)
Clinical and Supplemental Scales	Cognitive concerns	58.5	15.4	77 [89]	10 [11]	5.2***	0.55 (0.33, 0.78)
	Depression	58.0	15.8	72 [83]	15 [17]	4.7***	0.50 (0.27, 0.72)
	Suicidal ideation	54.2	21.5	74 [85]	13 [15]	2.6**	0.28 (0.06, 0.49)
	Post-traumatic stress	55	14.1	74 [85]	13 [15]	1.8*	0.19 (0.02, 0.41)
	Social anxiety	52.0	12.3	81 [93]	6 [7]	1.5	0.16 (0.05, 0.37)
	Anxiety	51.3	10.3	83 [95]	4 [5]	1.2	0.13 (−0.09, 0.34)
	Manic activation	50.8	11.8	81 [93]	6 [7]	0.6	0.07 (−0.14, 0.28)
	Psychosis	47.5	11.7	83 [95]	4 [5]	−1.7*	0.21 (0.003, 0.42)
	Alcohol problems	47.1	11.0	83 [95]	4 [5]	−2.5**	0.27 (0.05, 0.48)
	Drug problems	48	11.7	83 [95]	4 [5]	−2.5**	0.27 (0.05, 0.48)
	Severe aggression	46.4	11.5	84 [97]	3 [3]	−2.9**	0.31 (0.09, 0.53)
	Paranoid ideation	45.6	8.0	85 [98]	2 [2]	−5.2***	0.55 (0.33, 0.78)
	Antisocial behaviour	44.6	8.3	85 [98]	2 [2]	−6.0***	0.65 (0.41, 0.88)
	Grandiosity	43.1	7.7	87 [100]	0 [0]	−8.4***	0.90 (0.65, 1.15)
	Psychosocial functioning	41	8.8	87 [100]	0 [0]	−9.6***	1.03 (.77, 1.29)

^*^p < 0.05, **p < 0.01, ***p < 0.001

**Fig. 3:**
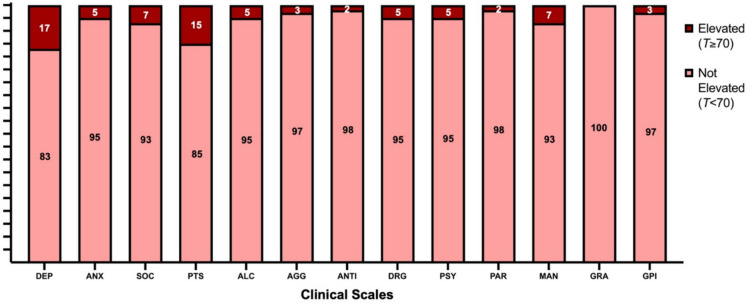
Percentage of the AE cohort reporting ‘elevated’ vs ‘not elevated’ levels of psychopathology on clinical scales. Data are based on a sample of 87 patients. *AGG* severe aggression, *ALC* alcohol problems, *ANTI* antisocial behaviour, *ANX* anxiety, *DEP*  depression, *DRG* drug problems, *GPI* general psychopathology index, *GRA* grandiose ideation, *MAN* manic activation, *SOC* social anxiety, *PAR* paranoid ideation, *PSY* psychosis, *PTS* posttraumatic stress

**Fig. 4:**
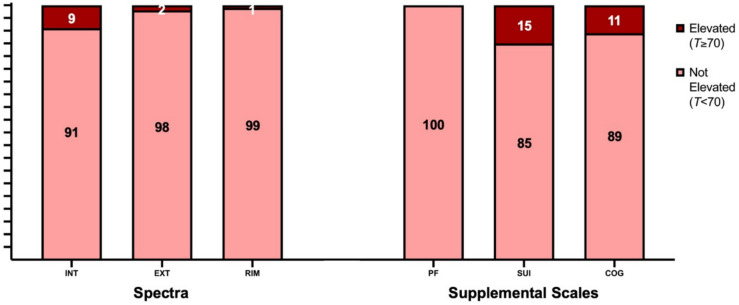
Percentage of the AE cohort reporting ‘elevated’ vs ‘not elevated’ levels of psychopathology on spectra and supplemental scales. Data are based on a sample of 87 patients

SPECTRA *T*-scores categorised symptom severity as ‘elevated’ and ‘not elevated’ in Figures 3, 4. Three percent of participants exhibited elevated Global Psychopathology. Among the three core spectra, Internalising symptoms had the highest proportion of patients scoring in the extremely elevated range (*T *≥ 70; 9%). Among the clinical scales, Depression had the highest percentage of patients classified as extremely elevated (17%), followed by Post-Traumatic Stress (15%).

### SPECTRA and AE subtype

We analysed only three subtypes—NMDAR AE (*n* = 21), LGI-1 AE (*n* = 17), and Seronegative AE (*n* = 42)—since these subtypes each included at least 10 participants. Relative to normative data, seronegative and NMDAR patients exhibited significantly elevated Cognitive Concerns, Depression, and Post‑Traumatic Stress, whereas no significant differences were observed in the LGI1 group (see Supplementary Tables 1–3).

In terms of subgroup comparisons, significant differences were revealed among subtypes in Anxiety (*H* = 10.4, *df* = 2, *p* = 0.005), Post-Traumatic Stress (*H* = 8.8, *df* = 2, *p* = 0.013), Severe Aggression (*H* = 10.3, *df* = 2, *p* = 0.006), Cognitive Concerns (*H* = 12.8, *df* = 2, *p* = 0.002), and Internalising Symptoms (*H* = 6.3, *df* = 2, *p* = 0.043).

Follow-up Mann–Whitney *U* tests indicated that the seronegative group exhibited significantly higher levels of Cognitive Concerns (*U* = 74, *Z* = 3.4, *p* = 0.001, *d* = 0.36), Anxiety (*U* = 78, *Z* = 3.3, *p* = 0.001, *d* = 0.35), Post-Traumatic Stress (*U* = 93, *Z* = 2.9, *p* = 0.004, *d* = 0.31), Internalising Symptoms (*U* = 102, *Z* = 2.6, *p* = 0.003, *d* = 0.28), Depression (*U* = 115, *Z* = 2.3, *p* = 0.001, *d* = 0.25), Severe Aggression (*U* = 123, Z = 2.3, *p* = 0.022, *d* = 0.24), and Global Psychopathology (*U* = 114, *Z* = 2.3, *p* = 0.042, *d* = 0.27) compared to the LGI1 group; as well as worse Severe Aggression (*U* = 109, Z = 2.7, *p* = 0.002, *d* = 0.29) and Cognitive Concerns (*U* = 123, Z = 2.1, *p* = 0.003, *d* = 0.23) than the NMDAR AE group. On the other hand, the NMDAR subtype significantly worse outcomes than those with the LGI1 subtype for Post-Traumatic Stress (*U* = 50, *Z* = 2.3, *p* = 0.004, *d* = 0.24). Overall, seronegative AE was associated with the most severe and widespread psychopathology. See Supplementary Figs. 1–5 for the distribution of *T*‑scores for each subgroup across the SPECTRA scales. Also see Supplementary Tables 1–3 for the descriptive statistics, overall classification scores, and results of one-sample *t*-tests comparing SPECTRA scores of the AE subgroup to normative data.

### SPECTRA and psychiatric comorbidities in AE

#### Comparison of patients with versus without psychiatric comorbidities

We compared SPECTRA scores between patients with and without psychiatric comorbidities. Patients with pre-existing psychiatric comorbidities (“pre-onset”, *N* = 20) reported significantly worse psychopathology compared to those without comorbidities (*N* = 59) in the domains of Post-Traumatic Stress, Depression, Social Anxiety, Cognitive Concerns, and Suicidal Ideation. The pre-onset group also exhibited significantly higher levels of internalising symptoms and a greater overall global burden of psychopathology. When considering all patients with psychiatric comorbidities (*N* = 28) versus those without, this pattern of heightened symptom severity persisted. A subgroup of patients who developed psychiatric comorbidities after onset (“post-onset”, *N* = 8) also showed elevated psychopathology compared to those without comorbidities in the same domains bar one (i.e., Depression). Note, there were no significant differences in SPECTRA scores between the pre-onset and post-onset groups (*p* > 0.05) (Table 3).

**Table 3:** Independent samples *t*-test results comparing SPECTRA scores between patients with psychiatric comorbidities versus those without comorbidities

		Pre-onset ( +) vs (−)		Post-onset ( +) vs (−)		Combined ( +) vs (−)	
		*t*	Cohen’s *d* (95% CI)	*t*	Cohen’s *d* (95% CI)	*t*	Cohen’s *d* (95% CI)
SPECTRA Indices	Internalising	3.1**	1.00 (0.46, 1.53)	1.9*	0.89 (0.13, 1.64)	3.5***	0.93 (0.46, 1.40)
	Reality Impairing	0.4	0.10 (−0.40, 0.61)	0.3	0.11 (−0.63, 0.85)	0.4	0.10 (−0.35, 0.55)
	Externalising	0.5	0.11 (−0.39, 0.62)	1.2	0.50 (−0.24, 1.24)	1.0	0.23 (−0.22, 0.68)
	Global	2.6**	0.72 (0.20, 1.24)	2.0*	0.81 (0.05, 1.56)	3.1**	0.74 (0.28, 1.20)
Clinical and Supplemental Scales	Post-Traumatic Stress	3.3***	1.04 (0.51, 1.57)	2.7*	1.32 (0.54, 2.09)	4.1***	1.07 (59, 1.55)
	Depression	2.8**	.90 (0.37, 1.42)	0.9	0.29 (−0.45, 1.03)	2.8**	0.72 (0.25, 1.18)
	Social Anxiety	2.8**	0.75 (0.23, 1.27)	2.3*	0.83 (0.07, 1.58)	3.3***	0.77 (0.31, 1.23)
	Anxiety	2.6**	0.77 (0.25, 1.29)	2.2*	0.94 (0.18, 1.69)	3.2***	0.80 (0.33, 1.26)
	Cognitive Concerns	2.1*	0.56 (0.04, 1.07)	0.9	0.33 (−0.41, 1.07)	2.1*	0.50 (0.04, 0.95)
	Suicidal Ideation	2.0*	0.71 (0.19, 1.22)	1.6	0.89 (0.13, 1.64)	2.5**	0.70 (0.24, 1.16)
	Psychosis	1.2	0.30 (−0.21, 0.81)	0.8	0.26 (−0.48, 1.00)	1.3	0.29 (−0.16, 0.74)
	Antisocial Behaviour	1.0	0.25 (−0.26, 0.76)	1.6	0.87 (0.12, 1.62)	1.7*	0.42 (−0.04, 0.87)
	Alcohol Problems	−0.9	0.17 (−0.34, 0.68)	1.0	0.45 (−0.29, 1.19)	0.1	0.02 (−0.43, 0.47)
	Drug Problems	−0.8	0.15 (−0.36, 0.65)	0.5	0.11 (−0.85, 0.63)	−0.8	0.14 (−0.59, 0.31)
	Severe Aggression	0.6	0.15 (−0.36, 0.66)	1.4	0.51 (−0.24, 1.25)	1.2	0.26 (−0.20, 0.71)
	Paranoid Ideation	0.3	0.08 (−0.43, 0.58)	0.3	0.11 (−0.63, 0.85)	0.3	0.08 (−0.37, 0.53)
	Manic Activation	0.2	0.06 (−0.44, 0.57)	0.04	0.01 (−0.73, 0.75)	0.2	0.05 (−0.40, 0.50)
	Grandiosity	0.2	0.06 (−0.44, 0.57)	0.5	0.17 (−0.57, 0.91)	0.4	0.09 (−0.36, 0.54)
	Psychosocial Functioning	−0.1	0.02 (−0.48, 0.53)	1.0	0.37 (−38, 1.11)	1.3	0.13 (−0.58, 0.32)

The pre-onset group includes patients with pre-existing psychiatric diagnoses prior to AE onset; the post-onset group consists of patients diagnosed with psychiatric disorders after AE onset; and the third subgroup encompasses both of these groups combined

^*^p < .05, **p < .01, ***p < .001

### SPECTRA and clinical outcome measures

#### SPECTRA and mRS

The median mRS was 2 (IQR: 1.0–2.0). We analysed mRS scores using two dichotomised outcome categories: “Good” (≤ 2) versus “Poor” (> 2) and “Good” (≤ 1) versus “Poor” (> 1). In the first category, majority (84%) of the patients were classified to have “good” outcomes (mRS ≤ 2), while 16% scored above 2; in the second category, approximately a third (35%) of the patients had “good” outcomes (mRS ≤ 1). For the first category, patients with “poor” outcomes demonstrated greater symptom burden compared to those with “good” outcomes in three domains: Antisocial Behaviour (*t*(10.72) = 2.4, *d* = 1.06, 95% CI 0.35, 1.76), Depression (*t*(11.61) = 2.13, *d *= 0.82, 95% CI 0.13, 1.51), and Externalising Symptoms (*t*(11.70) = 1.9, *d *= 0.74, 95% CI 0.05, 1.42). One other domain approached significance: Psychosocial Functioning (*t*(13.80) = 1.7, *d *= 0.54, 95% CI 0.15, 1.22). For the second category, no significant differences between patients with “good” and “poor” outcomes were found.

#### SPECTRA and CASE

The median CASE score was 2 (IQR: 0.0–3.0). CASE scores demonstrated a significant, moderate, positive correlation with Global Psychopathology (*rho* = 0.3, *d* = 0.70). Among the clinical scales, significant positive correlations were observed with Cognitive Concerns (*rho* = 0.4, *d* = 0.95), Depression (*rho* = 0.4, *d* = 0.82), Anxiety (*rho* = 0.3, *d* = 0.68), Severe Aggression (*rho* = 0.3, *d* = 0.63), Social Anxiety (*rho* = 0.3, *d* = 0.61), and Psychosis (*rho* = 0.3, *d* = 0.61). For the SPECTRA indices, CASE scores were positively correlated with Internalising Symptoms (*rho* = 0.4, *d* = 0.75) and Externalising Symptoms (*rho* = 0.3, *d* = 0.65).

#### mRS and CASE scores as predictors of psychopathology

As seen in Table 4, a hierarchical linear regression model showed that the mRS accounted for only a small proportion of variance across SPECTRA domains. The mRS accounted for 8–26% of the variance in Depression, Severe Aggression, Antisocial Behaviour, Psychosis, and Externalising symptoms; the inclusion of the CASE score moderately enhanced the model for Anxiety, Social Anxiety, Post-Traumatic Stress, Cognitive Concerns, and Internalising symptoms.

**Table 4:** Hierarchical linear regression results examining the contribution of mRS and CASE scores to SPECTRA scores

		mRS		CASE		CASE + mRS			mRS + CASE		
		*R*^*2*^	*B (L-U)*	*R*^*2*^	*B (L-U)*	*R*^*2*^	Δ*R*^*2*^	*B (L-U)*	*R*^*2*^	Δ*R*^*2*^	*B (L-U)*
SPECTRA Indices	Internalising	0.02	1.46 (−1.42–4.35)	0.10*	2.12 (0.46–3.79)	0.13	0.03	3.51 (0.94–6.08)	0.13	0.13*	−3.01 (−7.27–1.26)
	Externalising	0.08*	2.93 (0.31–5.55)	0.03	1.16 (−0.45–2.78)	0.08	0.05	−0.45 (−2.92–2.02)	0.08	0.08	3.50 (−0.61–7.61)
	Reality-Impairing	0.03	1.58 (−0.92–4.08)	0.02	0.90 (−0.60–2.41)	0.03	0.01	0.42 (−1.94–2.78)	0.03	0.03	1.05 (−2.87–4.96)
	Global	0.04	2.26 (−0.58–5.10)	0.11	2.26 (0.62–3.90)	0.12	0.01	2.95 (0.38–5.52)	0.18	0.18*	−1.50 (−5.78–2.77)
Clinical and Supplemental Scales	Depression	0.26*	4.18 (0.16–8.20)	0.14**	3.66 (1.34–5.98)	0.14	0.002	4.20 (0.57–7.84)	0.14	0.14*	−1.17 (−7.22–4.87)
	Anxiety	0.13	1.31 (0.32– −1.30)	0.09*	1.83 (.32–3.35)	0.11	0.02	1.17 (0.62–5.32)	0.11	0.11*	−2.47 (−6.37–1.43)
	Social Anxiety	0.02	1.97 (−1.42–5.36)	0.09*	2.44 (.47–4.4)	0.11	0.02	3.70 (0.64–6.75)	0.11	0.11*	−2.74 (−7.81–2.34)
	Post-Traumatic Stress	0.00	−0.25 (−3.69–3.19)	0.03	1.38 (−0.67–3.42)	0.08	0.05	3.61 (0.50–6.73)	0.08	0.08	−4.85 (−10.03–−0.33)
	Alcohol Problems	0.003	0.66 (−2.22–3.54)	0.002	−0.27 (−2.00–1.47)	0.02	0.02	−1.38 (−4.08–1.32)	0.02	0.02	2.42 (−2.06–6.91)
	Severe Aggression	0.10*	3.06 (0.67–5.44)	0.09*	1.75 (0.31–3.20)	0.11	0.02	0.83 (−1.41–3.07)	0.11	0.11*	2.00 (−1.73–5.73)
	Antisocial Behaviour	0.09*	2.54 (0.42–4.66)	0.03	0.89 (−0.43–2.20)	0.09	0.06	−0.68 (−2.67–1.32)	0.09	0.09*	3.40 (0.08–6.71)
	Drug Problems	0.01	1.11 (−1.39–3.61)	0.02	0.12 (−1.40–1.63)	0.02	0.02	−0.96 (−3.31–1.40)	0.02	0.02	2.33 (−1.58–6.24)
	Psychosis	0.09*	3.61 (0.65–6.57)	0.07*	1.87 (0.05–3.66)	0.30	0.06	0.47 (−2.33–3.26)	0.09	0.09*	3.02 (−1.63–7.66)
	Paranoid Ideation	0.04	1.77 (−0.51–4.06)	0.02	0.73 (−0.66–2.13)	0.04	0.02	−0.20 (−2.36–1.96)	0.04	0.04	2.03 (−1.56–5.63)
	Manic Activation	0.02	1.70 (−1.68–5.08)	0.01	0.84 (−1.20)	0.02	0.01	0.15 (−3.05–3.34)	0.02	0.02	1.52 (−3.79–6.83)
	Grandiosity	0.002	0.41 (−1.75–2.58)	0.01	0.43 (−0.87–1.73)	0.01	0.001	0.58 (−1.46–2.62)	0.01	0.01	−0.33 (−3.72–3.06)
	Cognitive Concerns	0.06	3.81 (−0.12–7.74)	0.15**	3.64 (1.39–5.90)	0.15	0.01	4.57 (1.05–8.09)	0.15	0.15**	−2.01 (−7.87–3.84)
	Psychosocial Functioning	0.003	−0.52 (−2.87–1.83)	0.001	−0.19 (−1.60–1.23)	0.003	0.002	0.12 (−2.10–2.34)	0.003	0.003	−0.67 (−4.36–3.02)
	Suicidal Ideation	0.01	2.51 (−2.95–7.97)	0.01	1.67 (−1.92–4.66)	0.01	0.003	0.51 (−4.64–5.67)	0.01	0.01	1.86 (−6.71–10.42)

Values represent unstandardised coefficients (*B*) and their 95% confidence intervals from the final step of the hierarchical regression model including both predictors

^*^p < 0.05, **p < 0.01

*CASE* clinical assessment scale in autoimmune encephalitis, mRS modified rankin scale

On the other hand, the CASE accounted for 7–15% of the variance in Depression, Anxiety, Social Anxiety, Severe Aggression, Psychosis, Cognitive Concerns, and Internalising symptoms; the inclusion of the mRS score weakly enhanced the model only for Antisocial Behaviour.

## Discussion

Overall, the results demonstrate that AE patients exhibit significantly elevated symptomatology within the Internalising spectrum (particularly in the domains of Depression and Post-Traumatic Stress), as well as in Suicidal Ideation and Cognitive Concerns. These findings are consistent with previous research, which has recognised mood disturbances as a prominent feature of the psychiatric sequalae of AE, affecting up to 70% of cases in the acute phase [26]. As demonstrated in our study, depressive symptoms may persist in the long term, potentially hindering overall recovery and significantly affecting patients’ quality of life. On the other hand, there is limited research on post-traumatic stress in AE, with only one study estimating that approximately 27% of AE cases experience post-traumatic stress [27]. Importantly, this prevalence may vary depending on the subtype or nature of the acute presentation; for instance, post-traumatic stress appears relatively uncommon in patients with profound amnesia during the acute episode (as seen in some cases of NMDAR AE) [28], as this likely prevents the consolidation of traumatic memories. It should be noted that the term ‘post-traumatic stress’ here refers to subjective distress reported in surveys and does not imply a formal diagnosis of post-traumatic stress disorder (PTSD). Still, given that AE is a life-threatening and often sudden neurological condition, it can be inherently traumatic for patients, resulting in significant emotional distress.

Moreover, our findings agree with previous studies which have associated cognitive outcomes to psychopathology in AE [29]. Indeed, cognitive impairment in AE can persist long after the initial diagnosis, often extending over months or even years [30]. These deficits—primarily poor memory and executive dysfunction—have been associated with reduced functional connectivity between the hippocampus and the medial prefrontal cortex, along with disruptions within the medial temporal lobe (MTL) network [31]. Many patients also report difficulty thinking and concentrating, and increased fatigability, which often impede the ability to return to work or school [30].

Suicidal ideation, although less commonly studied, has been reported in patients with AE. Prevalence rates range from 3.5 to 13% in other AE cohorts, with approximately 1.5% of patients dying by suicide [32–34]. In our study, our cohort of AE patients displayed significantly elevated levels of suicidal ideation compared to normative levels, with those having pre-existing psychiatric disorders being at a higher risk than those without. Previous research has identified suicidal ideation during the acute phase of AE [35, 36]. In contrast, far fewer studies have examined this psychopathology in the post-acute or chronic phases, with only one study estimating that approximately 17% of patients report suicidal ideation, suggesting that mood disorders with suicidal features may dominate the long-term psychiatric trajectory of AE survivors [37]. Our findings, drawn from a cohort assessed in the post-acute phase, indicate that the risk of suicidal ideation not only persists but may even intensify over time, underscoring the importance of ongoing psychiatric follow-up in this population. It is therefore crucial to assess suicidality in the acute phase to enable continuous monitoring of these symptoms, and further investigate the underlying factors contributing to suicidal risk in AE patients.

In terms of AE subtype, the seronegative group exhibited significantly higher levels of psychopathology compared to the LGI and NMDAR groups. This finding diverges from the existing literature, which has predominantly associated psychiatric syndromes to other AE subtypes, particularly NMDAR AE. While some studies have reported associations between seronegative AE and psychiatric symptoms such as depression [38], anxiety [39], and early psychosis [18, 19], the evidence remains limited. One study found that approximately 76% of their cohort of 147 patients with seronegative AE exhibited psychiatric symptoms, though this was not described in detail [3]. A plausible explanation for our findings may be the heterogeneity of the seronegative group. In the absence of a clearly defined antibody mechanism, diagnosis is often delayed and treatment pathways may be less targeted. As a result, patients with seronegative AE may experience a more protracted course of illness and greater long-term burden of psychiatric and cognitive symptoms. By contrast, antibody-defined syndromes such as NMDAR and LGI1 AE are more readily recognised and treated earlier with disease-specific immunotherapies, which may mitigate some of the these complications. Given the variability in psychiatric presentations across AE subtypes, future research should systematically document their distinct psychopathological profiles.

Finally, our findings indicate that the modified Rankin Scale (mRS) explains only a small proportion of the variance in psychopathology, particularly in Externalising symptoms and Depression. This aligns with prior research suggesting that the mRS has limited sensitivity in capturing post-stroke outcomes related to cognitive functioning and affective disturbances, especially in patients with more severe impairment [40, 41]. When added to the model, the CASE score provided more explanatory power, specifically in Internalising symptoms and Cognitive Concerns, indicating that it may offer a more sensitive measure of psychopathology. Indeed, in isolation, the CASE score had explained a greater proportion for a broader range of psychopathology symptoms (Depression, Anxiety, Social Anxiety, Severe Aggression, Psychosis, Cognitive Concerns) and overall Internalising symptoms. The inclusion of the mRS score weakly enhanced the model only for Antisocial Behaviour. The enhanced sensitivity of the CASE score could be attributed to its more comprehensive assessment of cognitive and emotional domains. Additionally, patients with “poor” outcomes (mRS > 2) had significantly higher levels of Depression and Externalising symptoms compared to those with “good” outcomes. However, when a more lenient mRS cut-off (≤ 1 vs > 1) was applied, this distinction between groups was no longer observed—suggesting that more severe disability is meaningfully associated with these psychological outcomes. These findings underscore that the mRS, as a relatively crude measure of functional outcome, is unable to detect the nuances of AE-related psychopathology. In contrast, the CASE, alongside more targeted assessment tools, may be better suited to identifying psychiatric sequelae in this population.

Overall, our findings indicate that psychopathology symptoms may persist in the post-acute phase of AE, highlighting the need for ongoing assessment and management of psychopathology in this population. However, several limitations must be acknowledged. First, we did not include repeated measures to compare time points and observe psychopathology symptoms over time, so it remains unclear whether the observed symptoms in our cohort fluctuate, improve, or worsen across different stages of recovery. Second, although informative, our sample size may limit the generalisability of these findings, particularly given the heterogeneity of AE subtypes and individual disease courses. This heterogeneity also precluded definitive conclusions about subtype-specific differences and limited the feasibility of adequately powered subgroup analyses. Expanding cohort sizes in future studies will be essential to robustly characterise psychiatric manifestations across AE subtypes. Lastly, this study relied solely on self-reported questionnaires to assess psychopathology which cannot establish formal psychiatric diagnoses. While such measures provide valuable insight into patient-perceived symptoms, they may not capture the positive psychological adjustment often observed in AE patients during clinical follow-up, especially relative to other neurological conditions such as multiple sclerosis [42], stroke [43], or traumatic brain injury [44]. Interpretation is further constrained by the absence of direct comparison groups, and self-report alone lacks the depth and contextualisation offered by clinician-administered assessments. Taking these findings in isolation risks overestimating the prevalence or severity of distress, including suicidality. Future research should incorporate clinician-rated assessments to validate and complement self-reported data, and adopt longitudinal designs to elucidate the trajectory of post-acute psychopathology and potential predictors of post-acute symptomatology. Collaboration with caregivers such as family members may provide additional insight into behavioural and cognitive changes that patients may not fully recognise or report. Finally, broader implementation of routine screening tools in clinical practice could facilitate early detection and intervention, ultimately improving long-term outcomes for AE patients.

This study highlights the significant burden of psychopathology symptoms among individuals with AE and the need for systematic, multidisciplinary approaches to assessment and management. While the mRS and CASE scores provide valuable clinical insights, their primary focus on functional outcomes may overlook the complexity and persistence of long-term psychiatric sequelae in AE. We emphasise the need for more sensitive and comprehensive assessment tools that better capture the full spectrum of patients’ psychological experiences. Future research and clinical practice should prioritise the integration of patient-reported outcomes, active caregiver involvement, and collaborative care models. Such approaches would provide a more nuanced understanding of psychopathology symptoms in AE and support more tailored, effective interventions to enhance long-term patient well-being.

## Supplementary Information

Below is the link to the electronic supplementary material.

Supplementary file1 (DOCX 363 KB)

## Data Availability

Raw data can be made available upon reasonable request. Please contact the corresponding author.
