# Supplementary material for: Subjective psychiatric symptoms in post-acute autoimmune encephalitis: findings from the Australian autoimmune encephalitis consortium
Source: J Neurol. 2025 Sep 29;272(10):665. doi: 10.1007/s00415-025-13353-0 (PMC12479715; doi:10.1007/s00415-025-13353-0)
Supplement: Supplementary file 1 — Supplementary file1 (DOCX 363 KB) [file 415_2025_13353_MOESM1_ESM.docx]

**Supplementary Tables**

**Supplementary Table 1.** Descriptive Statistics, Overall Classification Scores, and One-Sample *t*-test Results Comparing SPECTRA Scores of NMDAR AE Subgroup to Normative Data (*T*=50) across Spectra Indices, Clinical Scales, and Supplemental Scales.

|  |  |  |  | *Classification, N [%]* | |  |  |
| --- | --- | --- | --- | --- | --- | --- | --- |
|  |  | ***M*** | ***SD*** | ***‘Not Elevated’***  ***T<70*** | ***‘Elevated’***  ***T≥70*** | ***t*** | **Cohen’s *d* (95% CI)** |
| Spectra Indices | Internalising | 55.7 | 12.3 | 19 | 2 | 2* | .47 (-.01-.93) |
|  | Externalising | 43.7 | 6.5 | 21 | 0 | -4.3*** | .97 (.42-1.5) |
|  | Reality Impairing | 46 | 9.2 | 21 | 0 | -.2* | .45 (.03-.92) |
|  | Global | 51.4 | 10.3 | 21 | 0 | .6 | .13 (-.32-.58) |
| Clinical and Supplemental Scales | Depression | 58.4 | 17.3 | 16 | 5 | 2.1* | .48 (.001-.95) |
|  | Post-Traumatic Stress | 56.1 | 14.5 | 17 | 4 | 1.8* | .42 (-.05-.89) |
|  | Cognitive Concerns | 55.4 | 14.4 | 20 | 1 | 1.6* | .38 (-.09-.84) |
|  | Suicidal Ideation | 58.1 | 22.1 | 17 | 4 | 1.6 | .37 (-.10-.83) |
|  | Social Anxiety | 53.4 | 13 | 20 | 1 | 1.1 | .26 (-.20-.72) |
|  | Anxiety | 50.7 | 9.6 | 21 | 0 | .3 | .07 (-.38-.52) |
|  | Psychosis | 49.3 | 17.2 | 19 | 2 | -.2 | .04 (-.41-.49) |
|  | Drug Problems | 48.3 | 18.6 | 20 | 1 | -.4 | .09 (-.36-.54) |
|  | Manic Activation | 48.8 | 5.8 | 20 | 1 | -.5 | .11 (-.56-.34) |
|  | Severe Aggression | 44.7 | 6.8 | 21 | 0 | -3.4** | .78 (.25-1.28) |
|  | Grandiosity | 42.5 | 8.8 | 21 | 0 | -3.7*** | .85 (.32-1.37) |
|  | Paranoid Ideation | 44.8 | 5.8 | 21 | 0 | -.4*** | .89 (.35-1.42) |
|  | Psychosocial Functioning | 38 | 9.7 | 21 | 0 | -5.4*** | 1.24 (.63-1.83) |
|  | Antisocial Behaviour | 42.2 | 5.2 | 21 | 0 | -6.5*** | 1.49 (.82-2.13) |
|  | Alcohol Problems | 43.6 | 2.8 | 21 | 0 | -.10.1* | 2.31 (1.43-3.18) |

*Note.* **p*<.05, ***p*<.01, ****p*<.001.

**Supplementary Table 2.** Descriptive Statistics, Overall Classification Scores, and One-Sample *t*-test Results Comparing SPECTRA Scores of LGI1 AE Subgroup to Normative Data (*T*=50) across Spectra Indices, Clinical Scales, and Supplemental Scales.

|  |  |  |  | *Classification, N [%]* | |  |  |
| --- | --- | --- | --- | --- | --- | --- | --- |
|  |  | ***M*** | ***SD*** | ***‘Not Elevated’***  ***T<70*** | ***‘Elevated’***  ***T≥70*** | ***t*** | **Cohen’s *d* (95% CI)** |
| Spectra Indices | Internalising | 50 | 10.4 | 16 | 1 | 0 | .00 (-.51-.51) |
|  | Externalising | 45.4 | 8.8 | 16 | 1 | -2* | .52 (-1.06-.03) |
|  | Reality Impairing | 46.4 | 10.7 | 16 | 1 | -1.3 | .34 (-.19-.85) |
|  | Global | 46.1 | 10.1 | 16 | 1 | -1.5 | .38 (-.15-.90) |
| Clinical and Supplemental Scales | Depression | 52.9 | 15.1 | 16 | 1 | .7 | .19 (-.32-.70) |
|  | Cognitive Concerns | 51.7 | 15.5 | 16 | 1 | .4 | .11 (-.40-.62) |
|  | Manic Activation | 50.9 | 14.9 | 15 | 2 | .2 | .06 (-.45-.57) |
|  | Suicidal Ideation | 50.6 | 14 | 15 | 2 | .2 | .04 (-.46-.55) |
|  | Social Anxiety | 47.2 | 13.2 | 17 | 0 | -.8 | .21 (-.30-.72) |
|  | Psychosis | 47.3 | 12.7 | 16 | 1 | -.8 | .21 (.31-.72) |
|  | Alcohol Problems | 47.8 | 8.6 | 17 | 0 | -1 | .26 (-.26-.77) |
|  | Paranoid Ideation | 47.2 | 10.8 | 16 | 1 | -1 | .26 (-.26-.77) |
|  | Post-Traumatic Stress | 46.9 | 8.7 | 17 | 0 | -1.4 | .35 (-.18-.87) |
|  | Anxiety | 46.3 | 9.5 | 16 | 1 | -1.5 | .39 (-.15-.91) |
|  | Drug Problems | 46.1 | 8.3 | 16 | 1 | -1.8* | .50 (.07-.1.00) |
|  | Severe Aggression | 45.6 | 7.6 | 17 | 0 | -2.3* | .58 (.02-1.12) |
|  | Antisocial Behaviour | 42.8 | 7.7 | 17 | 0 | -3.6*** | .93 (-.31-1.53) |
|  | Grandiosity | 42.7 | 7.4 | 17 | 0 | -3.8*** | 1.00 (-.35-1.59) |
|  | Psychosocial Functioning | 40.1 | 9.9 | 17 | 0 | -3.9*** | 1.01 (.37-1.62) |

*Note.* **p*<.05, ***p*<.01, ****p*<.001.

**Supplementary Table 3.** Descriptive Statistics, Overall Classification Scores, and One-Sample *t*-test Results Comparing SPECTRA Scores of Seronegative AE Subgroup to Normative Data (*T*=50) across Spectra Indices, Clinical Scales, and Supplemental Scales.

|  |  |  |  | *Classification, N [%]* | |  |  |
| --- | --- | --- | --- | --- | --- | --- | --- |
|  |  | ***M*** | ***SD*** | ***‘Not Elevated’***  ***T<70*** | ***‘Elevated’***  ***T≥70*** | ***t*** | **Cohen’s *d* (95% CI)** |
| Spectra Indices | Internalising | 55.5 | 10.7 | 39 | 3 | 2.2* | .34 (.03-.65) |
|  | Externalising | 47.7 | 13.8 | 41 | 1 | -4.1*** | .63 (.29-.96) |
|  | Reality Impairing | 47 | 8.4 | 42 | 0 | -2.4** | .36 (.05-.67) |
|  | Global | 51.6 | 11.4 | 39 | 3 | -.9 | .14 (-.16-.45) |
| Clinical and Supplemental Scales | Cognitive Concerns | 61.3 | 15.8 | 36 | 6 | 4.6*** | .71 (.37-1.05) |
|  | Depression | 59 | 14 | 35 | 7 | 4.2*** | .65 (.32-.98) |
|  | Post-Traumatic Stress | 53 | 15.5 | 37 | 5 | 1.9* | .29 (-.02-.60) |
|  | Anxiety | 45.6 | 22.6 | 39 | 3 | 1.4 | .21 (-.09-.52) |
|  | Suicidal Ideation | 50.4 | 21.2 | 37 | 5 | -.1 | .14 (-.17-.44) |
|  | Severe Aggression | 47.8 | 12.2 | 40 | 2 | -.8 | .12 (-.19-.42) |
|  | Alcohol Problems | 48.5 | 14.3 | 38 | 4 | -.7 | .10 (-.20-.41) |
|  | Manic Activation | 50.7 | 14 | 39 | 3 | 1.0 | .05 (-.25-.36) |
|  | Social Anxiety | 50.6 | 13.3 | 38 | 4 | .3 | .05 (-.26-.35) |
|  | Drug Problems | 47.2 | 10.3 | 40 | 2 | -1.8* | .27 (-.04-.58) |
|  | Psychosis | 47.4 | 9.1 | 41 | 1 | -2* | .29 (-.02-.60) |
|  | Antisocial Behaviour | 46.2 | 10.5 | 39 | 3 | -2.3* | .36 (-.05-.67) |
|  | Paranoid Ideation | 45.6 | 8 | 41 | 1 | -3.6*** | .55 (.22-.87) |
|  | Grandiosity | 43.6 | 7.8 | 42 | 0 | -5.4*** | .83 (.47-1.17) |
|  | Psychosocial Functioning | 42.3 | 8.4 | 42 | 0 | -6.0*** | .92 (.56-1.28) |

*Note.* **p*<.05, ***p*<.01, ****p*<.001.

**Supplementary Figures**


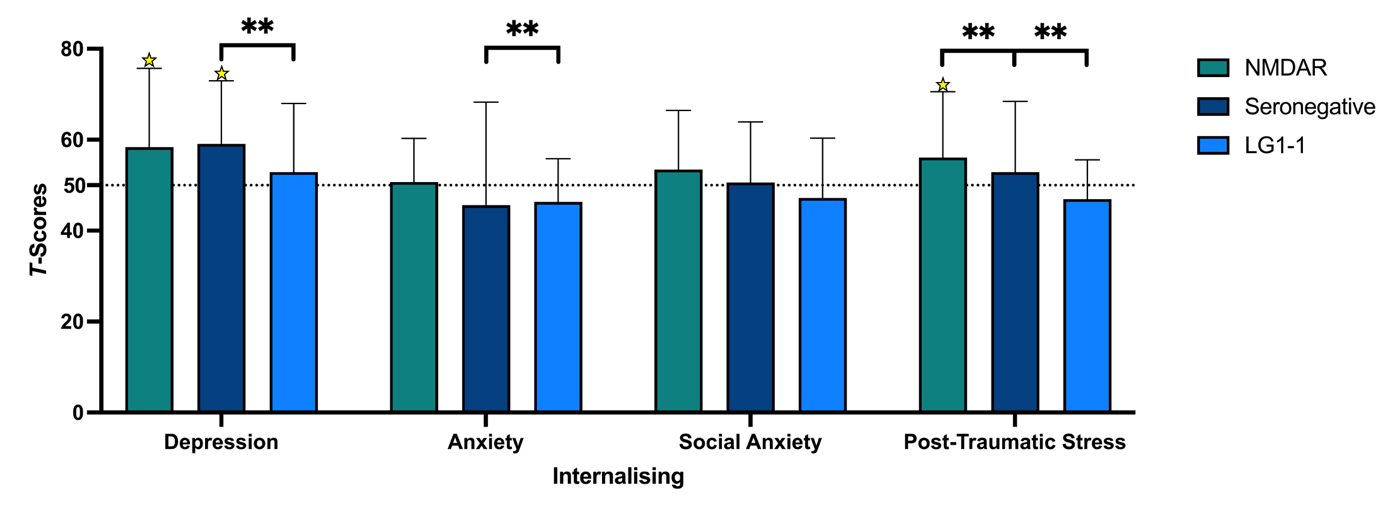


**Supplementary Figure 1.** Distribution of SPECTRA Internalising Scale *T*‑Scores. The horizontal line at *T*=50 represents the normative mean. Significant subgroup comparisons are indicated by **p*<.05 and ***p*<.01. Yellow stars denote scales with significantly elevated scores compared to normative data.


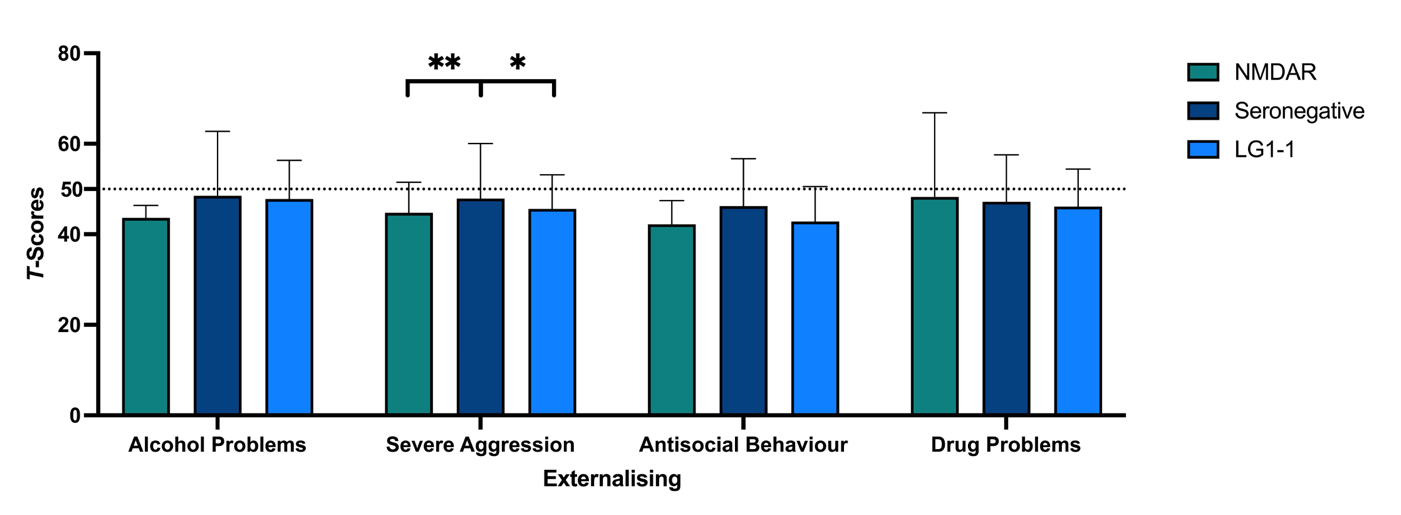


**Supplementary Figure 2.** Distribution of SPECTRA Externalising Scale *T*‑Scores. The horizontal line at *T*=50 represents the normative mean. Significant subgroup comparisons are indicated by **p*<.05 and ***p*<.01. Yellow stars denote scales with significantly elevated scores compared to normative data.


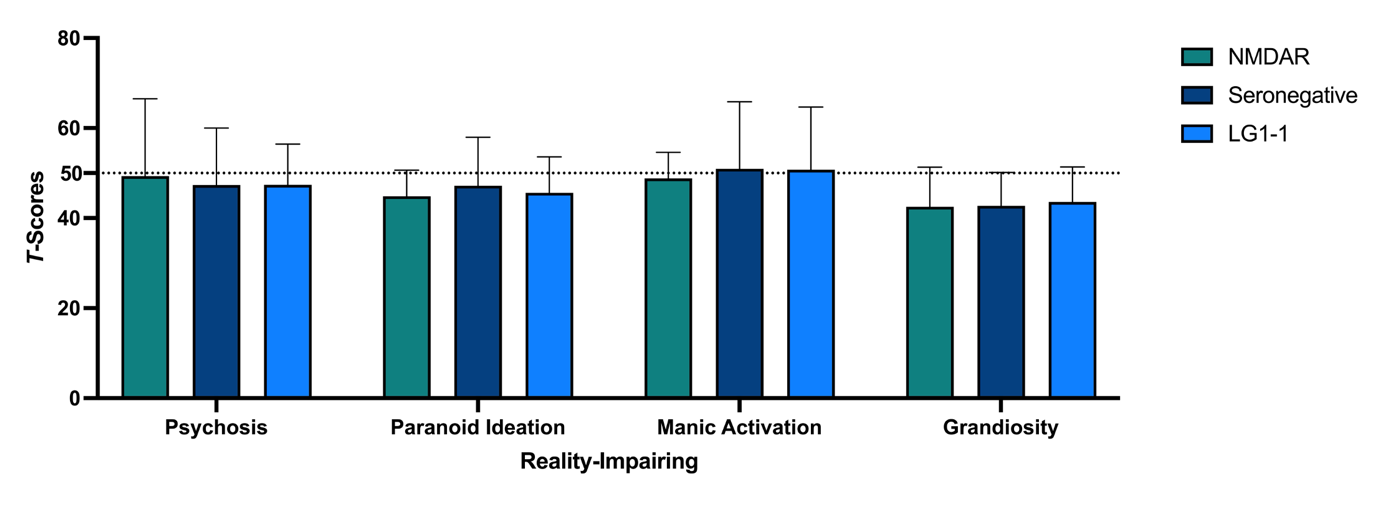


**Supplementary Figure 3.** Distribution of SPECTRA Reality-Impairing Scale *T*‑Scores. The horizontal line at *T*=50 represents the normative mean. Significant subgroup comparisons are indicated by **p*<.05 and ***p*<.01. Yellow stars denote scales with significantly elevated scores compared to normative data.


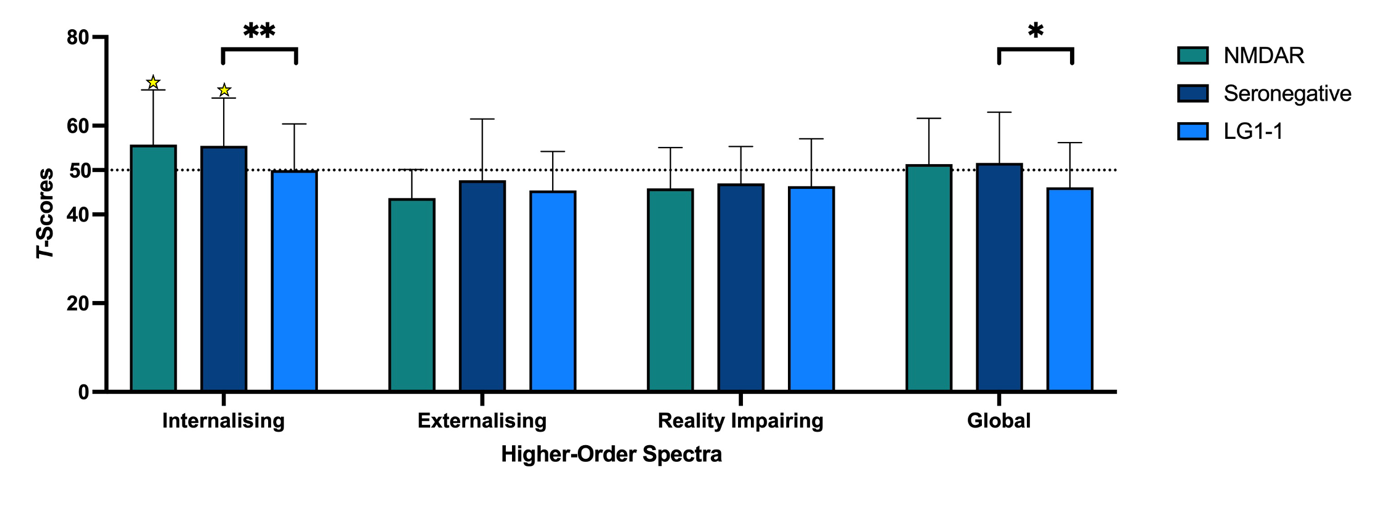


**Supplementary Figure 4.** Distribution of SPECTRA Higher-Order Spectra *T*‑Scores. The horizontal line at *T*=50 represents the normative mean. Significant subgroup comparisons are indicated by **p*<.05 and ***p*<.01. Yellow stars denote scales with significantly elevated scores compared to normative data.


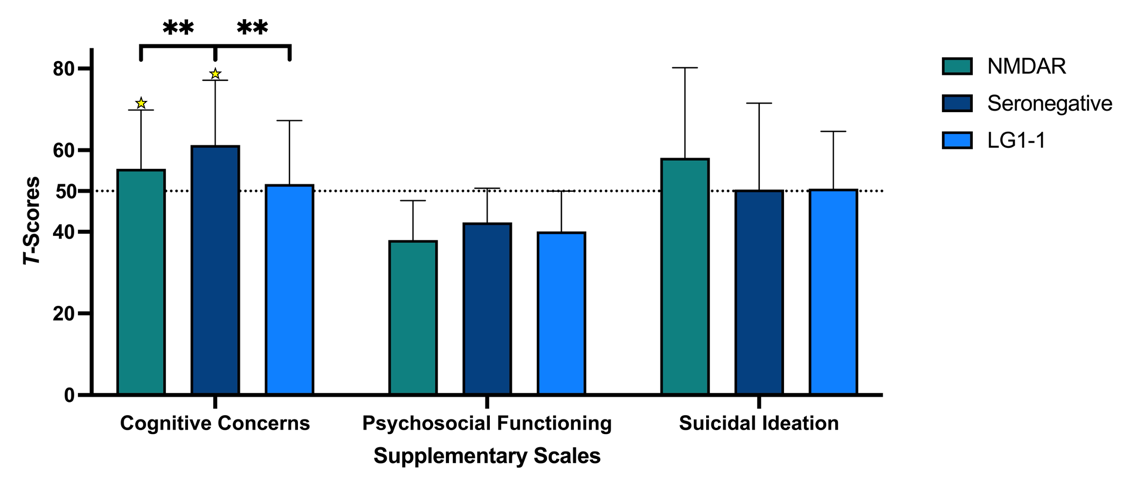


**Supplementary Figure 5.** Distribution of SPECTRA Supplementary Scale *T*‑Scores. The horizontal line at *T*=50 represents the normative mean. Significant subgroup comparisons are indicated by **p*<.05 and ***p*<.01. Yellow stars denote scales with significantly elevated scores compared to normative data.
